# Treatment of Refractory Syndrome of Inappropriate Antidiuresis With Sodium-Glucose Cotransporter-2 Inhibitor

**DOI:** 10.1016/j.aed.2025.09.013

**Published:** 2025-09-30

**Authors:** Nicole Chan, Aidar R. Gosmanov

**Affiliations:** 1 Division of Endocrinology, Department of Medicine, Albany Medical College, Albany, New York; 2 Section of Endocrinology, Stratton VA Medical Center, Albany, New York

**Keywords:** syndrome of inappropriate diuresis, sodium, water homeostasis, SGLT-2 inhibitor

## Abstract

**Background/Objective:**

The objective of this report is to describe a patient with syndrome of inappropriate antidiuresis (SIAD) who was successfully treated with empagliflozin monotherapy.

**Case Report:**

A 67-year-old man with a history of squamous cell carcinoma of the larynx and hypothyroidism was evaluated for persistent hyponatremia and diagnosed with SIAD. His serum sodium level remained below 130 mEq/L despite conventional therapy, including fluid restriction, furosemide, and sodium chloride tablets. He did not tolerate urea. Empagliflozin was added to his regimen resulting in improvement of serum sodium. Over time, furosemide was tapered and discontinued, followed by cessation of sodium chloride tablets. His serum sodium level normalized and remained stable on empagliflozin monotherapy without adverse effects.

**Discussion:**

Empagliflozin, a sodium-glucose cotransporter-2 inhibitor exerts its diuretic effects through inhibition of the corresponding channel at the proximal renal tubules, which is responsible for reabsorbing most filtered glucose. Inhibiting this channel, it produces an osmotic effect without the proportional sodium loss seen with loop diuretics. Conventional therapies for SIAD often yield only modest improvements in serum sodium with response rates below 50%. Notably, 2 recent proof-of-concept studies demonstrated that adding a sodium-glucose cotransporter-2 inhibitor to fluid restriction resulted in a statistically significant increase in serum sodium levels.

**Conclusion:**

This case highlights successful normalization of serum sodium levels using empagliflozin monotherapy, suggesting an alternative approach to addressing sodium and water dysregulation in SIAD. Its effectiveness seen in this case of refractory SIAD supports further investigation of empagliflozin in large-scale outpatient setting.

**Highlights**

• Syndrome of inappropriate antidiuresis (SIAD) is characterized by abnormal sodium and water homeostasis
• Conventional SIAD therapy is associated with low response rate
• Sodium-glucose cotransporter-2 inhibitors can be effective in treating refractory SIAD cases

**Clinical Relevance**

In patients with mild-to-moderate hyponatremia due to syndrome of inappropriate antidiuresis that persists despite standard of care therapy, addition of a sodium-glucose cotransporter-2 inhibitor may lead to significant improvement in serum sodium and even resolution of hyponatremia.

## Introduction

Syndrome of inappropriate antidiuresis (SIAD) is characterized by dysregulated antidiuretic hormone release, leading to impaired water excretion and hyponatremia. Common causes include medications, malignancies, pulmonary diseases, and human immunodeficiency virus infection.^1^ Because SIAD-induced hyponatremia is associated with adverse clinical outcomes,^2^^,^^3^ treatment efforts have focused on restoring water and salt homeostasis.

Fluid restriction remains the mainstay therapy; however, studies have shown only modest efficacy with serum sodium improvements and response rates often below 50%.^4^ Furthermore, combining fluid restriction with furosemide and/or sodium chloride therapy does not appear to significantly improve hyponatremia.^5^ Although urea has been used, high-quality trials are lacking to establish its efficacy and safety.^6^ There is no consensus on the optimal second-line therapy, and comparative data are limited. In that regard, pathophysiologic management of hyponatremia via administration of an oral vasopressin V2 receptor antagonist tolvaptan in heterogeneous population of ambulatory patients with chronic mild-to-moderate hyponatremia demonstrated plasma sodium reduction by 3 to 6 mmol/L.^1^ Despite its potential efficacy in patients with significant hypervolemic and euvolemic hyponatremia including SIAD as per the Food and Drug Administration label, the tolvaptan use as a second-line treatment is limited due to combination of factors such as its unknown efficacy in patients with hyponatremia of more than 30 days’ duration and baseline serum sodium level of >125 mEq/L, potential risk of rapid serum sodium overcorrection, lack of safety information with its long-term use, and high cost.^1^ Thus, effective management of SIAD remains challenging.

Sodium-glucose cotransporter-2 inhibitors (SGLT-2is) are approved for managing glycemic control and for reducing cardiovascular and kidney complications in people with type 2 diabetes. SGLT-2is block the reabsorption of filtered glucose from the tubular lumen in the proximal renal tubules, resulting in glucosuria and osmotic diuresis, mechanisms that may counteract the pathologic water retention seen in SIAD. One clinical trial showed positive trends in serum sodium following short-term treatment with the SGLT-2i empagliflozin; however, this approach remains off-label and requires further evidence before wider adoption.^7^ Here, we present a case of SIAD refractory to conventional therapy, in which initiation of empagliflozin led to normalization of serum sodium.

## Case Report

The patient was a 67-year-old man with a history of squamous cell carcinoma of the larynx, osteoporosis, and hypothyroidism secondary to neck radiation. He presented to the endocrinology clinic in 2023 for evaluation of progressive hyponatremia. An initial approach at his primary care clinic—fluid restriction to 1.2 L/d—did not lead to notable improvement in serum sodium, which remained consistently below 130 mEq/L. He had been diagnosed with stage III laryngeal cancer in January 2021 and had completed chemoradiation therapy successfully by May 2021. Since then, periodic clinical and radiological assessments had consistently shown no evidence of recurrence.

Evaluation in the endocrinology clinic ruled out adrenal insufficiency based on a morning serum cortisol level of 16.4 (reference, 5-25 μg/dL). His thyroid-stimulating hormone (TSH) level was 6.0 (reference, 0.5-5.0 mIU/L) consistent with subclinical hypothyroidism, which should not have decreased serum sodium levels. Other laboratory results included calculated serum osmolality of 256 mOsm/kg (reference, 275-295 mOsm/kg), which was confirmed by direct measurement; serum sodium level of 123 (reference, 135-145 mEq/L); urine sodium level of 46 (reference, >20 mEq/L); and urine osmolality of 334 mOsm/kg (reference, 50-1200 mOsm/kg). His lipid profile was normal. He did not have diabetes mellitus based on a fasting serum glucose level of 83 mg/dL and hemoglobin A1C level of 5.1%. Clinically, the patient was euvolemic and had normal blood pressure.

One year before his endocrinology evaluation, the patient was seen in the nephrology clinic, where he began urea at 30 mg twice daily; however, he discontinued it because of taste intolerance. He continued fluid restriction without improvement. In August 2023, at the endocrinology clinic, we initiated treatment with sodium chloride tablets (1 g twice daily) and furosemide (20 mg daily) (Fig.). Given the mildly increased TSH level noted on our initial evaluation, we re-educated the patient on the proper approach administration of levothyroxine regimen (88 μg daily), which led to normalization of TSH level to 2.58 mIU/L 2 months later. Although the serum sodium level increased slightly, it remained below 130 mEq/L. Given the lack of significant response, we introduced empagliflozin at 25 mg daily regimen in October 2023. Four weeks later, his serum sodium level improved by 4 mEq/L to 127 (Fig.); at that time to further optimize therapy, sodium chloride was increased to 2 g twice daily, and furosemide was reduced to 10 mg daily. In February 2024, the repeat serum sodium level was 130 mEq/L reassuring that current treatment was effective. Further serial sodium measurements showed a steady and sustained increase, with levels exceeding 135 mEq/L (Fig.).

**Fig:**
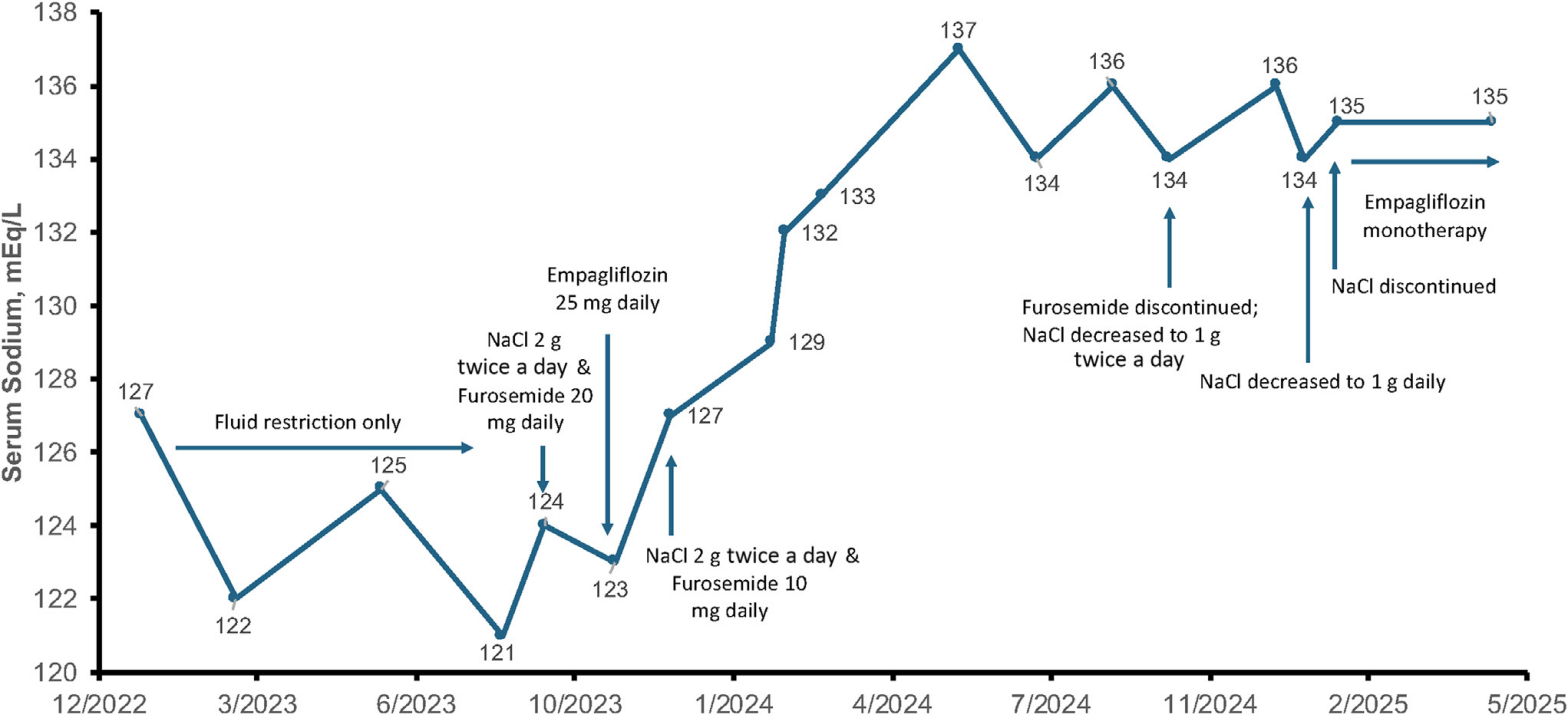
Serum sodium trend before and after empagliflozin initiation. *NaCl* = sodium chloride.

As the patient’s sodium improved and remained stable, from September 2024, we began tapering furosemide and sodium chloride. Subsequently, furosemide was discontinued, and sodium chloride was reduced to 1 g twice daily. By December 2024, sodium chloride was further reduced to 1 g daily, and in January 2025, it was discontinued entirely, leaving the patient on empagliflozin monotherapy. On that regimen, the serum sodium level remained stable and within the normal range (Fig.). Throughout treatment, the patient was continuously advised to maintain fluid restriction. He also remained biochemically euthyroid on a stable levothyroxine dose with TSH values ranging from 1.77 to 3.46 mIU/L. While on empagliflozin monotherapy for 4 months, his most recent serum sodium level was 135 mmol/L (Fig.) with corresponding serum osmolality of 302 mOsm/kg, urine osmolality of 511 mOsm/kg, and random urine sodium level of 64 mEq/L.

Throughout the treatment course, the patient experienced no adverse effects. In addition to reviewing his medication refill history, adherence to SGLT-2i therapy was confirmed by several urinalysis results showing significant glucosuria in the setting of euglycemia.

## Discussion

We have herein described a patient with euvolemic hypo-osmolar hyponatremia diagnosed in the outpatient setting. Adrenal insufficiency was excluded as a potential cause. Initially, the patient had a mildly increased TSH level while on chronic thyroid hormone replacement, which we promptly corrected, rendering him euthyroid well before any significant improvement in his hyponatremia. This suggests that mild subclinical hypothyroidism was an unlikely contributor to his presentation. Indeed, recent studies indicate that even moderate-to-severe hypothyroidism is unlikely to play a significant role in the development of hyponatremia.^8^^,^^9^ Furthermore, because his squamous cell carcinoma of the larynx was in remission, he did not have paraneoplastic syndrome of inappropriate secretion of antidiuretic hormone. Taken together, we felt confident that our patient had SIAD in the absence of apparent endocrine or other reversible etiologies known to cause euvolemic hyponatremia.

Older adults, like our patient, are particularly susceptible to hyponatremia due to age-related changes in vasopressin regulation, the presence of comorbidities, and polypharmacy. Hyponatremia is associated with increased morbidity and mortality, with recognized risks including neurologic impairment and osteoporosis. Emerging evidence suggests that correcting hyponatremia may improve both cognition and bone health. Current treatments, however, have notable limitations in adherence, tolerability, and efficacy, whereas newer options—such as vasopressin receptor antagonists and protein supplementation—may not be suitable for all patients.^10^ Tolvaptan is approved by the Food and Drug Administration for the management of hyponatremia in patients with diagnosis of less than 30 days’ duration and whose serum sodium level is <125 mEq/L. The patient’s history of hyponatremia that predated endocrinology consultation along with lack of safety data on long-term use of the V2 vasopressin receptor antagonist led us to believe that the initiation of tolvaptan was not warranted. In this context, SGLT-2is represented a promising alternative, potentially better tolerated and well suited for older adults with multiple comorbidities.

Empagliflozin may promote effective diuresis through its action on SGLT-2, located in the proximal renal tubules and responsible for reabsorbing the majority of filtered glucose from the tubular lumen.^11^ By inhibiting SGLT-2, empagliflozin increases urinary glucose excretion creating an osmotic effect that draws water into the urine and facilitates its excretion. Importantly, this process does not result in a proportional loss of sodium, allowing serum sodium concentrations to remain stable or increase. By contrast, furosemide, a loop diuretic, may be less effective in this context because of its mechanism of action as it inhibits tubular reabsorption of sodium in addition to water. This lack of selectivity for water excretion may explain why furosemide failed to achieve adequate correction of hyponatremia in this case, whereas empagliflozin demonstrated greater efficacy. Based on presented evidence of unsuccessful attempts to restore the patient’s serum sodium with standard of care approach that included fluid restriction, sodium chloride, and furosemide and attainment of eunatremia only following the initiation of empagliflozin, we submit that observed recovery from hyponatremia was likely due implementation of SGLT-2 inhibition rather than spontaneous resolution of SIAD.

Initial evidence for empagliflozin’s efficacy in SIAD comes from a randomized, double-blind, placebo-controlled trial conducted in Switzerland. This study included 88 hospitalized patients with syndrome of inappropriate antidiuretic hormone secretion–induced hyponatremia. Those who received empagliflozin at 25 mg daily alongside fluid restriction (1 L per 24 hours) showed a significantly greater increase in plasma sodium levels after 4 days than did patients who received placebo and fluid restriction.^7^ Another randomized, double-blind, placebo-controlled crossover trial evaluated empagliflozin at 25 mg daily in 14 patients in the outpatient setting with chronic syndrome of inappropriate antidiuretic hormone secretion–induced hyponatremia. In that study, patients with mild SIAD treated with an SGLT-2i for 4 weeks experienced a significant increase in the serum sodium level of 4.1 mmol/L from a baseline median of 131 mmol/L.^12^ In our patient, the time to resolution of hyponatremia following empagliflozin initiation was longer than that reported by Refardt et al,^12^ likely because of his lower baseline serum sodium as well as possible challenges with adherence to fluid restriction in real-world clinical practice. Nevertheless, both prior studies and this case highlight the potential of SGLT-2 inhibition as a reliable and effective therapeutic option for SIAD management.

## Conclusion

In this case of refractory SIAD, the patient achieved sustained normalization of serum sodium levels on long-term empagliflozin monotherapy, with discontinuation of adjunctive therapies and no adverse effects. This outcome aligns with emerging evidence supporting the potential of SGLT-2is in treating refractory SIAD. Given the high burden of hyponatremia in older adults and the limitations of current therapies, this case underscores the need to explore novel treatment strategies. A successful response, as demonstrated here, may help expand options for patients who are unresponsive or intolerant to conventional therapy. Further studies are warranted to establish the long-term safety and efficacy of SGLT-2is—and empagliflozin specifically—in this setting.

## Disclosure

The authors have no conflicts of interest to disclose. A.R.G. is employee of the U.S. Department of Veterans Affairs and opinions expressed in this manuscript is author’s personal opinions and do not necessarily represent the opinion of the Department of Veterans Affairs.
